# Genetic Heterogeneity in Four Probands Reveals *HGSNAT*, *KDM6B*, *LMNA* and *WFS1* Related Neurodevelopmental Disorders

**DOI:** 10.3390/biomedicines12122736

**Published:** 2024-11-29

**Authors:** Behjat Ul Mudassir, Mujaddid Mudassir, Jamal B. Williams, Zehra Agha

**Affiliations:** 1 Translational Genomics Laboratory, Department of Biosciences, COMSATS University, Islamabad 45550, Pakistan; fa16-pmg-001@isbstudent.comsats.edu.pk; 2 Rawalpindi Institute of Cardiology, Rawal Road, Rawalpindi 46000, Pakistan; mujaddidmudassir26@gmail.com; 3 Department of Psychiatry, Jacobs School of Medicine and Biomedical Sciences, State University of New York at Buffalo, Buffalo, NY 14203, USA

**Keywords:** cognitive diseases in four families, neurodevelopmental syndromic probands, harmful mutations in Pakistani families, mono-allelic and bi-allelic variants causing NDDs

## Abstract

**Background**: Neurodevelopmental disorders of genetic etiology are a highly diverse set of congenital recurrent complications triggered by irregularities in the basic tenets of brain development. **Methods**: We present whole exome sequencing analysis and expression characteristics of the probands from four unrelated Pakistani consanguineous families with facial dysmorphism, neurodevelopmental, ophthalmic, auditory, verbal, psychiatric, behavioral, dental, and skeletal manifestations otherwise unexplained by clinical spectrum. **Results**: Whole exome sequencing identifies a novel, bi-allelic, missense variant in the *HGSNAT* gene [NM_152419.3: c.1411G > A (p. Glu471Lys) exon 14] for proband family E-1 and a rare, bi-allelic, non-frameshift variant in the *KDM6B* gene [NM_001348716.2: c.786_791dupACCACC (p. Pro263_Pro264dup) exon 10] for proband family E-2, and a novel, mono-allelic, missense variant in the *LMNA* gene [NM_170707.4: c. 1328 A > G (p. Glu443Gly) exon 8] for proband family E-3 and an ultra-rare, mono-allelic, missense variant in the *WFS1* gene [NM_006005.3: c.2131G > A (p. Asp711Asn) exon 8] for proband family E-4. Protein modelling shows conformation and size modifications in mutated residues causing damage to the conserved domains expressed as neurocognitive pathology. **Conclusions**: The current study broadens the distinctly cultural and genetically inbred pool of the Pakistani population for harmful mutations, contributing to the ever-expanding phenotypic palette. The greatest aspirations are molecular genetic profiling and personalized treatment for individuals with complex neurological symptoms to improve their life activities.

## 1. Introduction

Neurodevelopmental disorders are triggered by an array of risk factors that can interrupt stages of development of the brain, and the cumulative effect of these risk factors determines the phenotype in adults [1]. Brain development is a greatly challenging process by which an array of neuronal and non-neuronal varieties of cells are created and incorporated into effective pathways in a structured way [2]. Several genes and mutations are linked to disruptions of critical neurodevelopmental pathways, indicating a diverse etiology for these conditions [3]. Genotype–phenotype connections are hard to pin down because of the presence of several genetic and environmental variables that impact the neurodevelopment. Most of the genes are associated with shared biological processes as identified by clinical and genetic studies exploring the ways by which individual mutations disrupt overlapping pathways and contribute to the onset of the neurodevelopmental disorder. Similarly, the comorbidity of more than one neurodevelopmental disorder in the same individual is observed very often [4]. Pedigrees with multiple individuals affected exhibit partial penetrance and phenotypical heterogeneity. The study of inherited NDDs has enormous promise for identifying the genetic origins and recognition of the risk factors. In contrast, the protective variables provide precise genotype–phenotype correlations to reveal major principles of phenotypical outcome, including gene vulnerability and mutational burden [5]. Vulnerable genes are less tolerant to disruptive events, and monogenic disorders originate with mutations bearing low selection rate in the general population. Genes, less vulnerable to the rare genetic disruptions, are frequently selected in the population for expression of normal phenotypes [6]. Multiple genetic factors like mutational load, dose sensitivity and epigenetic interaction contribute and correlate to the severity of symptoms in the affected individual [7,8]. Genetic studies with larger cohorts depict the co-occurrence of ID, ASD, DD, and seizures in patients with neurodevelopmental phenotypes where bi-allelic variations impact severely as compared to the mono-allelic. [9] Studies suggest monogenic NDDs as less frequent, to their heterogeneous or multifactorial/polygenic outcome. [10] The pathogenesis of NDDs discussed by the genetic analyses of global data explore the functional basis of the reported mutations [11].

From clinical, histopathological, and genetic data, the precise diagnosis of the complex syndromes has been possible. Genetic mutations with cognitive impairment, delayed development and motor retardation may present a case of differential diagnosis with neurodegenerative mechanisms and neurodevelopmental phenotypes [12]. The identification and characterization of phenotypes with respect to genotype is quite challenging to prevent inaccurate diagnosis [13]. Phenotype variability and technological advancements have increased our understanding of brain development cascade at the cellular level. Combined with more accurate tools, superior disease models and an increasing number of genetic variations linked to illness, we are far more likely to find disruptive pathways. Nonetheless, experimental, and conceptual hurdles lay ahead and require careful attention [14]. Significant studies on the genetics of NDDs showed the effects of mutations in more than 1500 genes [15], including bi-allelic and mono-allelic/de novo variants in *HGSNAT*, *KDM6B*, *LMNA* and *WFS1* genes [16,17,18,19]. The tremendously variable phenotypic canvas associated with genetic and molecular variations is the focus of this study.

We present here four unrelated consanguineous families with neurodevelopmental disorders as intellectual disability and developmental delay with comorbid epilepsy. Four unrelated probands with diverse facial features including dysmorphic characteristics, ophthalmic, dental, cardiac, and dermal complications have been observed by birth. These four probands bear behavioral and psychiatric disturbances as an important part of their personalities with variable expression. Clinically, these phenotypes were difficult to assign to any disease hence genetic testing was performed. Whole exome sequencing was performed to identify the pathogenic variants, by inhouse filtration pipeline and subsequent validation explored *HGSNAT*, *KDM6B*, *LMNA*, and *WFS1* related variants. These identified variants were analyzed functionally by in-silico protein modelling. Our study focusses on the biochemical pathways inside cellular organelles, mainly endoplasmic reticulum, lysosomes, and cytoplasm. The complex process of cellular proliferation, cellular migration, cell-to-cell communication, and strict balance of Ca^+2^ ions in the cells during neurodevelopment starts at the third week of the embryonic life and continues after the nineth week when the human brain reorganizes itself for maturity [20]. The population of the province Punjab, Pakistan, has its own distinct genetic makeup of highly inbred families where neurodevelopmental disorders have had a high prevalence rate in the past decade. Scarce genetic testing in remotely located areas due to the unavailability of funds in an economically poor society and generation after generation of consanguinity make these families’ genotypes unique with rare and novel mutations. The scanning of the gene pool of these families for potential risk variants is the requirement of the hour to provide counselling to avoid the birth of affected children in the future with neurodevelopmental phenotypes.

This study has significantly contributed to the global data, by the addition of novel, ultra-rare and rare genetic variants in *HGSNAT*, *KDM6B*, *LMNA* and *WFS1* genes for the first time in a Pakistani population that opens a new era for neurodevelopmental diseases, congenital anomalies, comorbid disorders, the effect of the presence or absence of family history, consanguinity, and personalized medicines to improve the condition of afflicted individuals.

## 2. Materials and Methods

### 2.1. Enrolment of Probands in Four Families

In the province of Punjab, Pakistan, remote areas of villages in north, west, south, and central regions were visited during field trips organized from 20 June 2021 to 30 November 2021 to explore the neurodevelopmental disorder families by research team of Translational Genomics Lab, COMSATS University, Islamabad. The aim was to identify the incidence and prevalence of neurodevelopmental phenotypes in geographically remote and less privileged population clusters to extend free genetic testing to the patients and counselling to their families about the diagnosis, management, and treatment of the diseases. Four unrelated consanguineous families (E-1, E-2, E-3, E-4) and probands (P-1, P-2, P-3, P-4) with rare phenotypes of intellectual disability and associated neurological, ophthalmic, skeletal, and dermal disorders were included in this study after proper diagnosis from clinicians at neurological and psychiatric department OPDs in hospitals attached with Rawalpindi Medical University, Rawalpindi. This study was approved by the ethical review board of Biosciences Department, COMSATS University, Islamabad, wide notification CIIT/Bio/ERB/17/50, dated 9 October 2017. The standards and norms of the research were in accordance with the Declaration of Helsinki ethical guidelines. Parents of the probands were informed about the study in detail verbally as well as by the distribution of questionnaires to obtain written consent for blood samples collection, whole exome sequencing and publication of the data.

Proband P-1 was a seven-year and one-month-old boy (IV-1) with severe intellectual disability (IQ < 40), developmental delay in speech, cognition and social interaction with bilateral retinal degeneration and seizures. Neurologists diagnosed him clinically as having a suspected neurodevelopmental syndrome, but his exact phenotype was not confirmed here. Genetic testing was required to ascertain the genomic variation for precise diagnosis.

Proband P-2 was a nine-year-old girl (IV-3) with intellectual impairment (IQ < 30), facial dysmorphism including a high arched forehead, widely spaced eyes, a broad nose, deep-set ears, microdontic, moody and stubborn with frequent epileptic seizures for the first year of her life.

Proband of P-3 was a sixteen-year-old boy (III-1) with severe intellectual impairment (IQ < 30), cardiac manifestation, diabetes mellitus, a cataract in the right eye, seizures, and skin atrophy on his back.

Proband P-4 was a nine-year-old girl (IV-1) with intellectual decline (IQ < 25), microcephaly and seizures by birth. She was drooling and smiling at everyone and could not recognize her blood relations.

**Figure 1:**
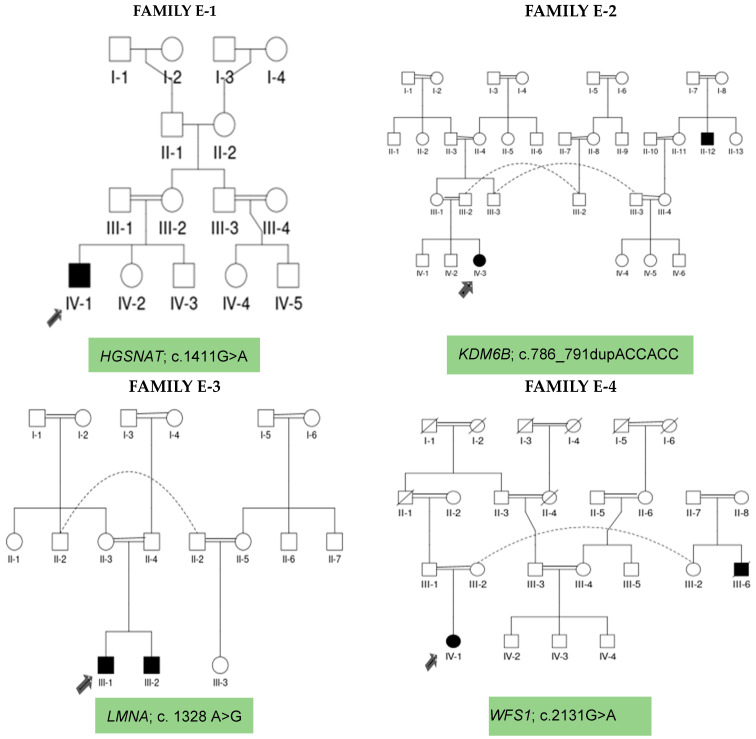
Pedigrees of families E-1, E-2, E-3 and E-4 showing affected members (shaded), while pointed arrows are directed towards the probands. Dotted lines indicate the displacement of family members due to marriage. Circles represent females, squares represent males and diagonal lines represent deceased members.

All four probands of these unrelated families were referred to Benazir Bhutto Hospital, Holy Family Hospital Rawalpindi, and Rawalpindi Institute of Cardiology for detailed medical examination. After recording clinicals tests at neurology, psychiatry, cardiology, pathology, dentistry, ophthalmology, and orthopedic clinics, these probands (P-1, P-2, P-3, P-4) were diagnosed as having neurodevelopmental and neurological syndromic conditions. The demographical and clinical manifestations were recorded in Table 1, while the inheritance pattern and family history were recorded as pedigrees in Figure 1.

The consultants suggested the families have genetic testing performed for confirmation of the diagnosis. Translational Genomics Research Group in collaboration with a team of consultants worked on the genetic identification of these four probands using whole exome sequencing.

### 2.2. Pedigree Analysis, Blood Sample Collection and DNA Extraction and Storage

The pedigrees of families E-1, E-2, E-3 and E-4 were kept as records of up to four to five previous generations, and a history of any CNS impairment was traced by interviewing the parents and close relatives. Pedigree E-1 did not show any history of neuro-disorder in the families, while pedigrees E-2, E-3 and E-4 depicted positive family history for neurodevelopmental disorders. Clinical symptoms of all four probands (P-1, P-2, P-3, P-4) were recorded, and 2.5 ml peripheral blood samples from the probands, their unaffected biological parents, siblings, and close relatives (Table 2) were collected in EDTA tubes. Further processing of these samples was carried out at a translational genomics lab for DNA extraction using a phenol-chloroform method. Extracted DNA was preserved at −20 °C for use in future experimentation.

### 2.3. Whole Exome Sequencing and Data Analysis

Whole exome sequencing of the preserved DNA samples was performed from December 2021 to February 2022 at Macrogen Inc. Seoul, Republic of Korea using an Illumina Nova Seq 6000 sequencer. Library preparation was carried out using an Agilent Sure Select Exome Capture kit following manufacturer’s instructions (Agilent Technologies, Santa Clara, CA, USA). Alignment of reads with reference genome hg38 was carried out using Burrows–Wheeler Aligner (BWA) software (v0.7.13). Variants Calling was performed with the Genome Analysis Toolkit (GATK), and resultant variants were annotated with Illumina Variant Studio v2.2. Received data in the form of VCF files for all four probands (P-1, P-2, P-3 and P-4) were uploaded to Franklin Genoox online software (v78.3) accessed on 15 March 2022 and resultant variants were subjected to downstream bioinformatics analysis by in-silico pathogenicity prediction tools Polyphen2, SIFT, Mutation Assessor, Mutation Taster and FATHMM. Prediction scores (Haploinsufficiency, triplosensitivity, missense Z-score, LOF probability score/DECIPHER values) were assessed to evaluate the gene–disease association. Evaluation of the variants was performed by the American College of Medical Genetics (ACMG) guidelines for human genetics syndromic disorders. The frequency of the alleles was examined in control population databases including 1000 Genomes, gnomAD and ESP6500 for rare variant identification and pathogenicity detection. Those variants which have more than 0.01 minor allele frequency in any of human genome databases (1000 Genomes Project, Genome Aggregation Database (gnomAD), Exome Sequencing Project 6500 (ESP6500), and database of single-nucleotide polymorphisms (dbSNP) were excluded from analysis. Resultant variants were filtered and prioritized by an inhouse pipeline [21] for the identification of variants associated with all four proband (P-1, P-2, P-3, P-4) phenotypes.

### 2.4. Validation of Variants and In Silico Protein Modelling

An inhouse pipeline identified variants in all four probands (P-1, P-2, P-3, P-4). To evaluate the segregation of the variants with the disease phenotypes in the family, genomic DNA extracted from the blood samples of unaffected parents, probands, siblings and their close relatives (Table 2) was analyzed by Sanger sequencing. Primers were designed using Primer 3 software (https://primer3.ut.ee) accessed on 11 May 2022. The exons containing the target variants were amplified through polymerase chain reaction following standard protocols. These genetic variants were validated by co-segregation analysis using conventional Sanger sequencing by ABI Prism 3130 Genetic Analyzer Applied Biosystems to generate Ab1 files. Ab1 files for each proband, their unaffected parents, affected/unaffected siblings and close relatives were uploaded and analyzed by Finch TV (v1.4) software. Reference sequences of the genes were downloaded from the Ensembl genome browser (Table 3 and Table 4). Protein modelling for the validated pathogenic genetic variants was carried out by HOPE and Alphafold2 online tools to determine the in-silico characteristics of defective protein structures. 

## 3. Results

### 3.1. Demographic/Clinical Characteristics of E-1, E-2, E-3, and E-4 Families

Family E-1 is a simplex family with no family history of any kind of NDDs in the previous four generations, while siblings (IV-2, IV-3) of proband P-1 were normal phenotypically. The seven-year and one-month-old proband (IV-1) was a boy with severe intellectual disability, developmental delay, bilateral retinal degeneration, and epileptic seizures. Clinically, he was suspected of having a neurodevelopmental disorder phenotype, while genetically, he was diagnosed as suffering from neurodegenerative disorder caused by a novel, bi-allelic missense variant in *HGSNAT* gene leading to the identification of Sanfilippo syndrome C disorder.

Family E-2 is a multiplex family with milestones of developmental delay and epilepsy in the affected members. Proband P-2 was a nine-year-old girl (IV-3) with intellectual impairment, frontal bossing, dental anomalies, depressive mood, and seizures. Her siblings (IV-1, IV-2) were normal in terms of phenotype, though there was a family history of intellectual impairment in the previous generation (II-12) of this family. Clinically, the proband (IV-3) was suspected of having neurodevelopmental disorder, which has been confirmed after genetic diagnosis. WES identified a rare, bi-allelic non-frameshift variant in the *KDM6B* gene.

Family E-3 is a simplex family with proband P-3, a sixteen-year-old boy (III-1) affected with intellectual impairment, cardiac manifestation, diabetes mellitus, a cataract in the right eye, epilepsy, and skin atrophy on his back. This family shows no positive history of NDDs in the five previous generations, while sibling (III-2) was affected by a NDD phenotype. Clinically, proband (III-1) was diagnosed as having a neurodevelopmental disorder. His genetic testing confirmed this, and WES identified a novel, mono-allelic missense variant in the *LMNA* gene.

Family E-4 was recruited as a trio. Proband P-4, a nine-year-old girl (IV-1), showed phenotypic manifestations of intellectual impairment, microcephaly, and epilepsy. There was a positive family history in previous generations (III-6) for cognitive impairment. Clinically, she (IV-1) was suspected of having a neurodevelopmental disorder, while her genetics confirmed the same diagnosis via WES, identifying an ultra-rare, mono-allelic missense variant in the *WFS1* gene. 

### 3.2. Whole Exome Sequencing Data Analysis and Variant Identification

**Figure 2:**
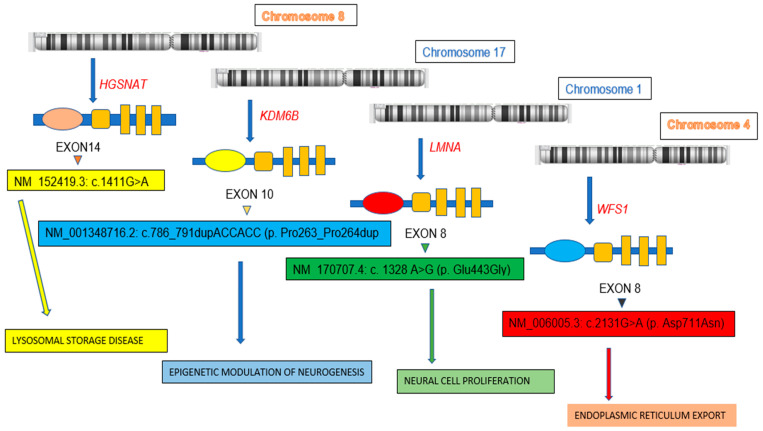
Diagrammatic representation of effects of *HGSNAT*, *KDM6B*, *LMNA* and *WFS1* gene variants on different processes of cellular functioning and neurodevelopment.

Samples of all four probands from families E-1 (IV-1), E-2 (IV-3), E-3 (III-1) and E-4 (IV-1) were subjected to whole exome sequencing analyses. Whole exome sequencing, variant identification of the obtained data, variant filtration and downstream analysis were performed by an inhouse pipeline. The process identified a novel, bi-allelic, missense variant in the *HGSNAT* gene [NM_152419.3: c.1411G > A (p. Glu471Lys) exon 14] for proband family E-1 and a rare, bi-allelic, non-frameshift variant in the *KDM6B* gene [NM_001348716.2: c.786_791dupACCACC (p. Pro263_Pro264du) exon 10] for proband family E-2, and a novel, mono-allelic, missense variant in the *LMNA* gene [NM_170707.4: c. 1328 A > G (p. Glu443Gly) exon 8] for proband family E-3 and an ultra-rare, mono-allelic, missense variant in the *WFS1* gene [NM_006005.3: c.2131G > A (p. Asp711Asn) exon 8] for proband family E-4 (Table 3). Physical, genetic, and biochemical characteristics at the level of sub-cellular organelles were reviewed and presented in Figure 2. These variants were subjected to validation by co-segregation analysis via the conventional Sanger sequencing of probands of all four families and their unaffected parents, siblings, and close relatives as a control.

### 3.3. Sanger Sequencing for Co-Segregation Analysis

**Figure 3:**
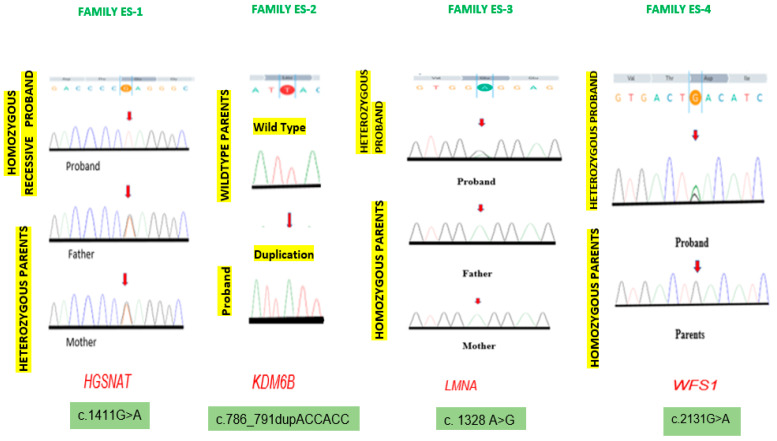
Graphical representation of co-segregation analysis of *HGSNAT*, *KDM6B*, *LMNA* and *WFS1* genetic variants in probands and unaffected parents.

The variants identified in all four probands were validated by Sanger sequencing (ABI Prism 3130 Genetic Analyzer Applied Biosystems) to generate Ab1 files, which were analyzed using Finch TV (v1.4) for the proband of each family. Reference sequences for the four genes were downloaded from the Ensembl genome browser. Co-segregation analysis of Sanger sequence electropherograms of probands, unaffected parents, siblings, and close relatives, mentioned in Table 2, (Figure 3) revealed it as denovo in families E-3 and E-4 (*LMNA*, *WFS1*) or inherited from the carrier parents in families E-1 and E-2 (*HGSNAT*, *KDM6B*). Sanger sequencing has confirmed the clinical diagnosis and genetic attributes of complex and unexplained phenotypes of the probands P-2, P-3 and P-4 in the three families, while P-1 was differentially diagnosed as a neurodegeneration case, as shown in Figure 3. This validation of the genetic variants explored the normal protein structure as disrupted, and the function was compromised as analyzed by in-silico protein modelling.

### 3.4. In-Silico Protein Modelling

HOPE and Alphafold2 protein modelling were performed, and the effects of the *HGSNAT* pathogenic variant on the structure and function of mutant protein were assessed. Wild-type residues were different from mutant residues in structure, which disturbs the conformation of the proteins. As these mutated residues are located at a highly conserved region, the mutations are damaging to the protein.

The *KDM6B* duplication changes the amino acid sequence, and there is a prominent difference in the sizes of the wild-type and mutant residues that might cause bumps in the mutant protein and disturb the normal structure and interactions.

The *LMNA* pathogenic variant affects the structure and function of the mutant protein. Mutant residue is smaller with a neutral charge and more hydrophobic as compared to the wild-type residue, which is negative. As the mutant residue is smaller in size, it cannot make the same hydrogen bond that the wild type does at a highly specialized region. It might disturb the domain and abolish the protein structure. The region of wild-type residue is highly conserved, and this mutation is damaging to the protein.

**Figure 4:**
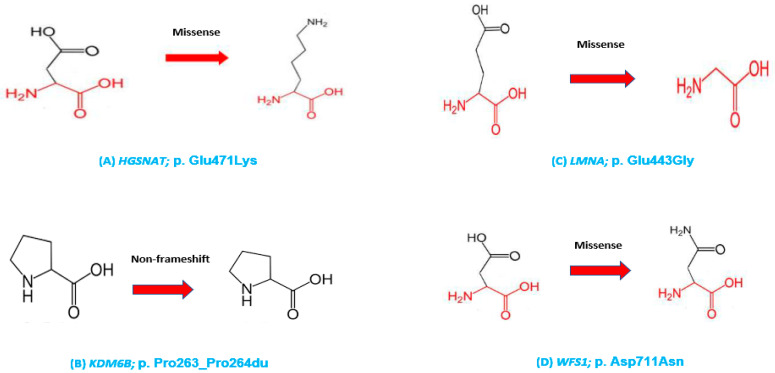
HOPE/Alphafold2 in-silico protein modelling results show the mutant protein residues (**A**–**D**) of the *HGSNAT*, *KDM6B*, *LMNA* and *WFS1* gene variants, respectively, that affect the structures and properties of proteins. The red arrows point towards mutant residue.

The pathogenic variant in the *WFS1* gene affects the structure and function of the mutant protein as the mutant residue charge is neutral as compared to the wild-type residue, which is negative. The mutant residue is located at a stretch of residues. The mutation is damaging to the protein due to the loss of interaction between the protein residues (Figure 4).

## 4. Discussion

We present two bi-allelic and two mono-allelic variants in a study cohort of four unrelated Pakistani families (E-1, E-2, E-3 and E-4) recruited from different areas of the Punjab province.

Proband family E-1, whole exome sequencing data analysis identified a bi-allelic pathogenic variant in the *HGSNAT* gene causing intellectual impairment and ophthalmic, psychiatric, behavioral, and severe neurological manifestations. The proband P-1 was initially diagnosed as having a suspected neurodevelopmental disorder. Genetic testing via WES and the identification of a novel pathogenic variant in the *HGSNAT* gene [22] led to the conclusion of a differential diagnosis [23,24], pertaining to be a very interesting finding in our cohort. It depicted the overlap of neurodevelopmental and neurodegenerative conditions, i.e., Sanfilippo syndrome C in the proband P-1.

Sanfilippo syndrome C, an inherited metabolic disorder, is a rare lysosomal storage disorder caused by mutations in the heparan--glucosaminide N-acetyltransferase *HGSNAT* gene, which results in heparan sulphate buildup [25]. Sanfilippo syndrome C is distinguished by significant neuropsychiatric symptoms as well as minor somatic signs [26]. The patients suffer from speech decline, neurological impairment, retinal degeneration, and hepatic and renal anomalies [19]. Large cohorts of Sanfilippo syndromic patients reveal recurrent as well as novel variants of the *HGSNAT* gene. These patients suffer from progressive neurodegeneration, dementia, and death in early adulthood [12,27,28]. There is currently no authorized therapy for Sanfilippo C syndrome. Non-syndromic retinitis pigmentosa and night blindness have been documented in certain individuals. The ocular phenotypes show decreased rods and cones in the retina, neuroinflammation, demyelination and early death [29]. These patients also suffer from autism spectrum disorder with delayed communication and behavior disturbances. To date, the data have not been well documented except for a few studies and research reviews around the globe. These describe genetic abnormality regarding the absence or significant reduction of enzymes involved in the breakdown of heparan sulphate (HS), resulting in HS accumulation primarily. The storage of other chemicals, as well as alterations in the expression of hundreds of genes and other abnormalities in organelles and metabolic processes in the cell, has been added secondarily, with tissue and organ malfunction causing severe symptoms. The neurological, cognitive, and behavioral impairments in CNS are extremely stressful for patients and their families [30]. The lack of a recognized therapy for Sanfilippo syndrome C care through a multidisciplinary approach is critical for treatment to improve patients’ quality of life. Efforts for an accurate diagnosis can optimize symptoms for patients’ care [31,32].

Denovo variants are the source of neurodevelopmental disorders as each monogenic NDD is unique and rare. The phenotype vs. genotype range of each morbid gene remains a big issue. OMIM describes heterozygous *KDM6B* mutations as a “neurodevelopmental disorder with coarse facies and mild distal skeletal abnormalities”. Cognitive abnormalities are found in the patients with *KDM6B* mutations, with wide differences in the entire phenotype. Coarse facies and distal skeletal deformities with other neurological characteristics have been observed in the proband of family E-2. Epigenetic changes are recognized to have an important role in neurogenesis, learning and memory. *KDM6B* produces polymers that interfere with expression throughout the development of the cerebral cortex of the forebrain subventricular region [13,16]. *KDM6B* inhibition can trigger neural stem cell differentiation that alters the development of numerous brain areas. *KDM6B* has been linked to the regulation of brain precursors [33] and formation of medial and lateral paraganglionic cells [34]. The deletion of *KDM6B* in the neuroectodermal differentiation pathway of humans leads to efficient induction in human pluripotent stem cells [16,35].

*LMNA*-related phenotypes are heterogeneous and diverse with muscular dystrophy, lipodystrophy, cardiomyopathy, skin lesions and frequent neurological and ophthalmic outcomes. This study provides the discovery of a novel, monoallelic, de novo variant in the *LMNA* gene as causative of intellectual disability, lipodystrophy, cardiac symptoms, skin atrophy, cataract, and diabetes mellitus [36] in the proband of family E-3. Various studies have reported neurological and neuropsychiatric impairments as linked with respect to *LMNA* gene variants [37]. Reported literature from Pakistani cohorts discusses the *LMNA* gene related to premature aging, while we report a novel variant which expands phenotypic expression of the laminopathies by the addition of neurological manifestations, lipodystrophy and hypotonia [38]. *LMNA*-associated genetic disorders have been investigated over the previous decade, with diverse categories including skeletal muscle disorders, lipodystrophy diseases, neuropathological disorders, and rapid aging disorders [17,39,40].

Proband of family E-4, whole exome sequencing identified a variant in the *WFS1* gene. *WFS1* genetic mutations cause a rare, autosomal, recessive neurogenetic condition affecting multiple organs and functioning. The syndrome is characterized by diabetes mellitus, optic degeneration, and hearing loss. Juvenile diabetes with declining vision and hearing in a child with developmental milestones characterizes the abnormal functioning of the *WFS1* gene [41,42]. Wolframin protein, encoded by *WFS1*, has been investigated as ER integrity and function, protein folding and cellular stress responses. It may also influence calcium homeostasis in ER and insulin secretion in pancreatic beta cells [43,44]. *WFS1* gene functioning is not completely understood. The researchers explained the functional significance in ER pathways, translational control, and protein degradation mechanisms. WFS1 is associated with mitochondria and neuronal complexes that affect the interaction and signaling of mitochondria [45]. The probable impact on cellular physiology explains the WFS1-related complex syndromic characteristics [46]. Frequently reported findings of heterozygous variants in the *WFS1* gene cause deafness and increased risk for diabetes type 2 [47,48].

The human brain requires cautious control of the regulation and expression of genes for proper functioning. Variations in genes that control the expression of associated proteins have been related to neurological, neurodevelopmental, and neurodegenerative disorders [49]. Neurodevelopmental disorders are an increasing clinical concern in contemporary society. Advanced tools for diagnosis have revealed an exceptionally complex architecture that comprises genetic mutations with varying frequencies in the population. A network composed of interrelated participants makes it challenging to establish solid genotype–phenotype relationships [50]. Additionally, individual habits may contribute to the nature of symptoms, triggering a wide range of interactions at the sub-cellular level that play an important part in linking genotypes with specific phenotypes. Identifying causative genetic variants can help design targeted therapeutics for more complex phenotypes, inducing a range of physiological implications [51]. The inbred population of Pakistan has the potential for underlying molecular consequences. Cellular pathways converge to an abnormal cascade under the pressure of common, rare and denovo genetic mutations in neurodevelopmental disorders in the consanguineous families [52]. This study explores the effects of impaired gene expression and disruption of biochemical processes inside sub-cellular organelles for the *HGSNAT*, *KDM6B*, *LMNA* and *WFS1* genes.

## 5. Conclusions

Advancements in the developmental research have expanded the concept of neurodevelopment. Independence, communication, reasoning, cognitive control, language, socializing and psychiatric and behavioral well-being depend on the correct functioning of the underlying genetic and biochemical processes of the brain. We present the role of *HGSNAT*, *KDM6B*, *LMNA* and *WFS1* genes in the neurodevelopment and neurocognitive domains expressed for metabolic and neural disorders. This study contributes to the etiology of complex phenotypes by the genetic control of sub-cellular organelles and explains the case of differential diagnosis in proband P-1, another milestone for researchers of low socio-economic countries.

### Limitations

We present a dataset demonstrating the challenging scenario of WES data analysis, which may implicate the methods used to discover genetic alterations for gene diagnostic research plans. This study explores possible restrictions in pathogenic variant identification as follows:

1. Whole exome sequencing (WES) is an effective way of detecting mutations for monogenic hereditary illnesses. Although rapid and precise, WES fails to detect mutations in about 35% of instances due to diverse genetic variants of unknown significance, variants that are in deep intronic regions and hence ignored by exome capture techniques. This is a methodological constraint that precludes the efficient detection of sequence modifications.
2. Complementary approaches, such as whole genome sequencing, may be used to aid in the discovery of disease-associated genetic changes.

## Figures and Tables

**Table 1:** Demographic description of families E-1, E-2, E-3 and E-4 and their probands.

Family ID	Ethnicity	Family Type	Proband Phenotype	Sibling Phenotype	Congenital Anomalies	Neurological Comorbidities	Family History for up to Five Previous Generations	Clinical Diagnosis	Genetic Identification
E-1	Punjabi	Simplex	ID	Normal	Retinal degeneration	Seizures	Negative	NDD	Neuro degenerative Disorder
E-2	Punjabi	Multiplex	ID	DD/Seizures	Tooth Decay Frontal Bossing	Seizures	Positive	NDD	NDD
E-3	Punjabi	Simplex	ID	Normal	DD, DM, Cataract, Cardiac anomaly, Skin atrophy	Seizures	Positive	NDD	NDD
E-4	Punjabi	Trio	ID		Microcephaly	Seizures	Positive	NDD	NDD

**Table 2:** Results of probands, parents, siblings and close relatives who participated in the study.

Members from Pedigrees	Family E-1	Family E-2	Family E-3	Family E-4	Tested by
Proband	IV-1 (+/+)	IV-3 (+/+)	III-1(+)	IV-1(+)	WES analysis
Parents	III-1, III-2 (+/−)	III-1, III-2 (+/−)	II-3, II-4 (−/−)	III-1, III-2 (−/−)	Co-segregation analyses by Sanger Sequencing
Siblings	IV-2, IV-3 (+/−)	IV-1, IV-2 (+/−)	III-2 (+)	Nil	
Close Relatives	IV-4, IV-5 (+/−)	III-3 (−/−)	II-1, II-2 (−/−)	IV-2, IV-3 (−/−)	

**Table 3:** Genomic attributes of probands of families E-1, E-2, E-3 and E-4 according to GRCh38/hg20.

Family ID	Proband Gender/Age	Gene	Exon	mRNA Variant	Protein Variant	Mutation Type	Mutation Effect
E-1	M/7years	*HGSNAT*	14	NM_152419.3: c.1411G > A	p. Glu471Lys	Homozygous	Missense
E-2	F/9 years	*KDM6B*	10	NM_001348716.2: c.786_791dupACCACC	p. Pro263_Pro264du	Homozygous	Non-frameshift
E-3	M/16 years	*LMNA*	8	NM_170707.4: c. 1328 A > G	p. Glu443Gly	Heterozygous	Missense
E-4	F/9years	*WFS1*	8	NM_006005.3: c.2131G > A	p. Asp711Asn	Heterozygous	Missense

**Table 4:** Prediction of pathogenicity scores for disease–gene association in the four unrelated probands.

Gene	DECIPHER Values				Pathogenicity Prediction Values						
	pHaplo	pTriplo	Missense Z-score	pLi	Polyphen2	SIFT	MT	FATHMM	DANN	MetaLR	Fit Cons.
*HGSNAT*	0.79	0.56	2.85	0.92	0.9 (Damaging)	0 (Del.)	1 (Del.)	−4.21 (Del.)	1 (Del.)	0.53 (Del.)	0.71 (Del.)
*KDM6B*	0.89	0.93	2.51	1	0.8	0	1	-4.5	1	0.56	0.8
*LMNA*	0.72	0.84	3.10	1	1 (Damaging)	0 (Del).	1 (Del.)	−4.87 (Del.)	1 (Del.)	0.94 (Del.)	0.71 (Del.)
*WFS1*	0.27	0.13	2.43	0	0.92 (Damaging)	0 (Del.)	0.97 (Del.)	−4.24 (Del.)	1 (Del.)	0.85 (Del.)	0.71 (Del.)

## Data Availability

Data are available within the manuscript.

## Abbreviations

NDD	neurodevelopmental disorder
ID	intellectual disability
DD	developmental delay
ER	endoplasmic reticulum
HS	heparan sulphate
CNS	central nervous system
BBH	Benazir Bhutto Hospital
RIC	Rawalpindi Institute of Cardiology
